# Muscle contraction mechanism based on single molecule measurements

**DOI:** 10.1007/s10974-012-9332-7

**Published:** 2012-12-01

**Authors:** Toshio Yanagida, Yoshiharu Ishii

**Affiliations:** 1 Graduate School of Frontier Biosciences, Osaka University, 6-2-3 Furuedai, Suita, Osaka 565-0874 Japan; 2 Center for Information and Neural Network, Suita, Osaka Japan; 3 Quantitative Biology Center, RIKEN, Suita, Osaka Japan

**Keywords:** Muscle contraction, Single molecule measurements, Brownian movement, Muscle model, Flashing ratchet model

## Abstract

Single molecule measurements have shown that a muscle myosin step is driven by biased Brownian movement. Furthermore, they have also demonstrated that in response to strain in the backward direction a detached myosin head preferentially attaches to the forward direction due to an accelerated transition from a weak binding to strong binding state. Because they are consistent with the original Huxley model for muscle contraction, we have built a model that describes macroscopic muscle characteristics based on these single molecule results.

## Introduction

Drs. Michael and Kate Barany were an excellent team in the world of muscle research. It is a testament to their work that their names will be long remembered.

More than 50 years ago, using then contemporary structural and physiological data, A.F. Huxley proposed a simple yet elegant model for muscle contraction that still remains relevant today (Huxley 1957). The model assumes (1) that the thermal motion of myosin around an equilibrium point acts as the motive force for sliding movement on actin filaments and (2) the attachment and detachment of myosin to an actin filament occur in an asymmetric manner in the direction of the actin and myosin filaments. H.E. Huxley later proposed a model after considering the structural changes of myosin from electron microscopy images and low-angle X-ray diffraction pattern data (Huxley 1969). Two years later, A.F. Huxley and Simmons showed that multiple cross bridges states are essential for explaining the dynamic changes in muscle that arise with tension recovery after a sudden alteration in muscle length during contraction (Huxley and Simmons 1971). The existence of multiple actomyosin states has been supported by data from the 3D-atomic structure of myosin II (Rayment et al. 1993a, b), biochemical experiments (Geeves and Holmes 1999), fiber measurements (Piazzesi et al. 2002, 2007), single molecule fluorescence measurements (Forkey et al. 2003) and single molecule AFM (Kodera et al. 2010).

New technologies and techniques for molecular biology, structural biology and single molecule measurements have since revealed the molecular basis for actomyosin function (Schliwa 2002). This includes the discovery of many myosin types and how they interact with actin at the single molecule level. For example, a number of studies in the 90s directly showed cyclical interactions between muscle myosin and actin and stepwise myosin movement (Finer et al. 1994; Molloy et al. 1995; Ishijima et al. 1994, 1998). Thus, the mechanism of the myosin step, especially for processive myosins such as myosin V and VI (Mehta et al. 1999; Rock et al. 2001), which function as single molecules or in conjunction with a small number of other myosin molecules when transporting an object a long distance without dissociating from actin filaments, has become better understood. The single molecule behavior of non-processive myosin, however, like the myosin in muscle is less clear, primarily because it coordinates in large numbers when acting on an actin filament. Therefore, to describe the single molecule behavior driving muscle contraction, we must clarify how individual molecules assemble and then coordinate to function. One approach is to measure single molecule behavior in intact muscle and compare this with the behavior observed in isolated molecules (Kaya and Higuchi 2010). Another is to build a mathematical model (Marcucci and Yanagida 2012). In this article, we consider single molecule experiments to describe two key actomyosin properties from the original Huxley model: Brownian movement of the muscle myosin (Kitamura et al. 1999) and the search-and-catch mechanism (Iwaki et al. 2009) and explain how a model that uses information about these properties from single molecule studies to describe muscle’s macroscopic behavior (Marcucci and Yanagida 2012).

## Brownian movement of muscle myosin

### Scanning probe microscopy

Manipulation techniques such as the laser trap and microneedle and fluorescence imaging have been previously used to monitor step-wise myosin movement (Finer et al. 1994; Molloy et al. 1995; Ishijima et al. 1994; Yildiz et al. 2003). Analysis of the interval time between steps has shown that the step-wise movement associates with the hydrolysis of single ATP molecules. The coupling between the step motion and ATP hydrolysis was directly confirmed by simultaneous measurements of single molecule myosin displacements and single molecule fluorescence observation of ATP turnover (Ishijima et al. 1998).

Soon thereafter, Brownian movement within a single muscle myosin step was detected (Kitamura et al. 1999). Usually step movement during the hydrolysis of a single ATP molecule occurs rapidly, within a few milli seconds, which means that the detection of the Brownian movement requires a high signal to noise ratio. When studying non-processive muscle myosin with the aforementioned techniques, an actin filament is manipulated while it interacts with a myosin molecule attached to a glass slide such that the proteins are prevented from diffusing away. However, a number of compliant elements in the system decrease the stiffness and dampen the myosin motion. We therefore have developed a scanning probe microscopy to directly measure a single myosin molecule instead of the actin filament (Ishijima et al. 1994; Kitamura et al. 1999). The stiffness of the experimental system increased from 0.05 to 0.2 pN/nm when an actin filament was manipulated to >1 pN/nm when a single myosin head was manipulated. Coincidently, the thermal fluctuations of the probe (i.e. noise of the system) decreased from 4–9 to <2 nm. The significant drop in noise thus enables scanning probe microscopy to resolve 10–20 nm myosin displacements.

Scanning probe microscopy measurement requires a new scanning probe be prepared each time an experiment is attempted. We prepared individual scanning probes of varying stiffness and attached single myosin molecules to them (Kishino and Yanagida 1988). In our scanning probe system the number of myosin molecules attached to a probe can be confirmed directly by measuring the fluorescence intensity and photobleaching behavior of the fluorescence spots (Funatsu et al. 1995). We have also established methods to minimize the number of unlabeled and photobleached molecules during preparation to strengthen our proof that single molecules are indeed observed (Kitamura et al. 2005; Nishikawa et al. 2007). In contrast, determining the number of myosin molecules in laser trap experiments, where a large number of beads are prepared in solution at one time, requires a statistical approach (Svoboda and Block 1994).

#### Brownian movement within a single step

**Fig. 1:**
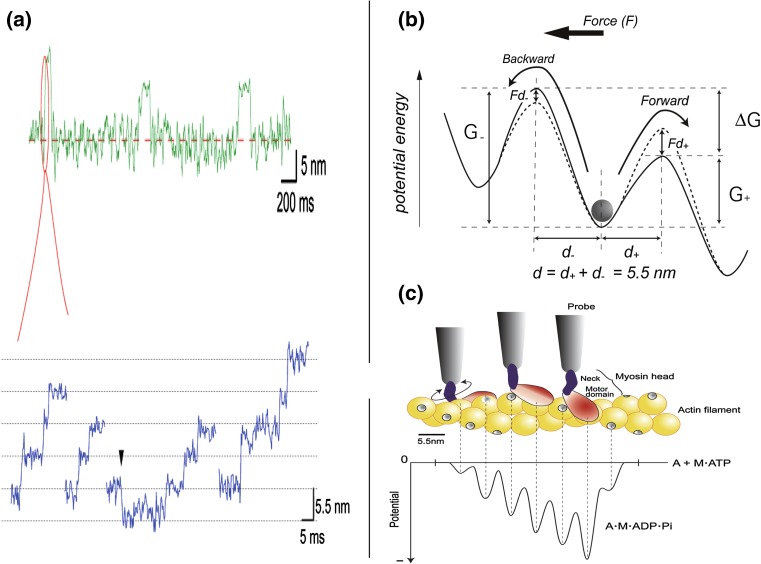
Biased Brownian movement of muscle myosin. **a** Displacement record of a single myosin molecule monitored by scanning probe microscopy. Steps hidden in the *above panel* can be resolved as rising phases when the time resolution is increased (*bottom panel*) (data are presented in Kitamura et al. (1999). **b** Energy landscape for biased Brownian movement of myosin along an actin filament. Local minima correspond to actin binding sites for myosin heads along an actin protofilament. Two potential energy paths are shown: the *solid line* represents a profile at zero external load, the *dashed line* when the system experiences an external load of F [Figure modified from Kitamura et al. (2005)]. **c** Energy landscape on an actin protofilament. The potential reflects the myosin and actin geometry in muscle. In single molecule experiments, the actin filament resembles a double stranded helix fixed onto a glass surface and myosin molecules are attached to a large scanning probe. Myosin heads rarely move to the other actin protofilament without detaching [Figure modified from Kitamura et al. (2005)]

The scanning probe method allows us to scrutinize the unitary steps made by myosin in conjunction with the hydrolysis of ATP. It can also be used to investigate the substeps within a single step (Fig. 1a). Computing the histogram of pairwise distances of all stepwise movements in the rising phase of a unitary step showed the size of these substeps to be ~5.5 nm, which corresponds to the distance between adjacent actin monomers on an actin protofilament. While most substeps occur in the forward direction, a small number were seen in the backward direction too. Although equal in size, the number of substeps varies randomly from one to five within a unitary step suggesting they do not tightly couple to ATP. Thus, the stochastic features of the stepping motion and step size strongly suggest that the myosin head walks or slides along the actin monomers using Brownian motion.

It has been reported that as the scanning probe stiffness increases, the number of substeps (especially the number of forward substeps) decreases without a change in size resulting in a smaller unitary step (Kitamura et al. 2005). This observation suggests the mechanism of Brownian myosin movement is unaffected by the load exerted on the myosin. From these data, we could obtain the velocity of myosin during the hydrolysis of single ATP molecules, which showed the relationship between velocity and load at the single molecule level resembles that in muscle.

Other myosin properties observed by the scanning probe method are consistent with other techniques. Yet the scanning probe method goes one above by being the only method that can observe substeps (Steffen et al. 2001; Ruff et al. 2001; Capitanio et al. 2006). This may be because the reaction process is significantly slowed in the scanning probe method (Fernandetz and Li 2004). It is also a superior method in that it constrains the motion of the myosin head to mimic the behavior in muscle (Kitamura et al. 2005). Therefore, scanning probe microscopy has the potential to provide a basis for the mechanical properties of muscle at the single molecule level.

### Biased Brownian step model

Because the majority of myosin substeps occur in one direction, we can assume the Brownian motion is biased (forward). In fact, at low force levels, the number of forward (N_f_) substeps is six times greater than the number of backward (N_b_) substeps. The Brownian movement of myosin can be represented as a periodic asymmetric potential (Fig. 1b). The activation energy of the forward and backward directions can be described as u_+_ + Fd_+_ and u_−_ − Fd_−_, respectively, where u_+_ and u_−_ are the heights of the maximum potential barrier at zero load (F = 0), and d_+_ and d_−_ are the characteristic distances. Assuming the Boltzmann energy distribution, the rates of the forward and backward directions at an external load, F, will be proportional to exp[−(G_+_ + Fd_+_)/k_B_T] and exp[−(G_−_ − Fd_−_)/k_B_T], respectively (Wang et al. 1998). Differences between the potential barriers for forward and backward substeps at F is given by ΔG − Fd = k_B_Tln (N_f_/N_b_), where ΔG = G_+_ − G_−_ and d = d_+_ + d_−_ = 5.5 nm. Experimental data describing the load dependence of the forward and backward substep ratio has shown that Brownian steps are biased by a potential energy of 2–3 k_B_T at zero load.

The above description follows a myosin head being attached to a large scanning probe that restricts its motion and orientation (Fig. 1c). Actin binding sites rotate along the filament, which is taut between two pedestals. Steric compatibility between the orientations of the myosin head and actin-binding site depends on their relative positions, resulting in a potential slope along the actin helical pitch. For example, if the binding site of the head faces the right side of an actin filament, then binding is favored to that side because any other direction would require the head to bend or rotate. Thus, the potential slope declines along the forward direction, which coincides with the actin half helical pitch. Such a potential restricts the number of substeps to no more than five.

## Brownian search-and-catch mechanism

Single molecule measurements have suggested that muscle operates by the Brownian search-and-catch mechanism, which assumes myosin heads undergo Brownian motion back and forth along the actin filament in the presence of ATP while they attach and detach rapidly (Dunn and Spudich 2007; Shiroguchi and Kinosita 2007; Iwaki et al. 2006). Eventually the myosin head binds strongly and preferentially to the forward direction, resulting in a transition from a weak binding to strong binding state, a transition accelerated by backward strain. This mechanism has been best explored in myosins V and VI (Mehta et al. 1999; Rock et al. 2001). When the two heads of myosin VI span the helical pitch of an actin filament, the front and rear heads are exposed to intra-molecular backward and forward strain, respectively. When ATP is bound to a rear head strained forward, the head detaches and undergoes Brownian movement while repeatedly and rapidly detaching and attaching. The head attaches to the forward direction more easily and then undergoes a transition to the strong binding state due to backward strain resulting in preferential binding to the forward direction. Because this mechanism has already been seen in multiple types of myosin, we argue it also applies to muscle myosin where the head is connected to rigid thick filament and strain can be applied. This has significant implications on the Huxley model, because the rate of attachment and detachment is assumed dependent on the direction of binding.

### Experimental evidence for strain-dependent transition from weak to strong binding states

**Fig. 2:**
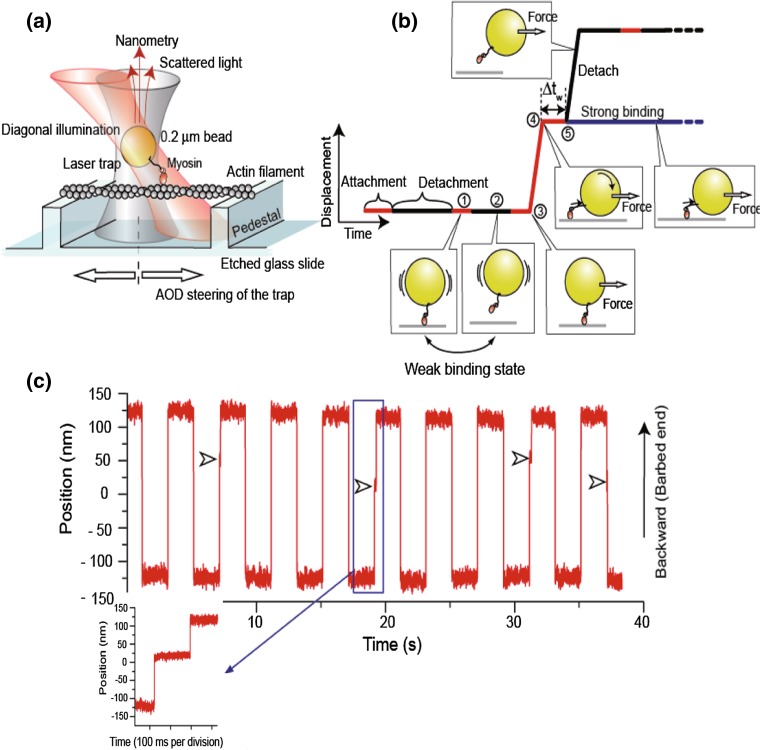
Experiments for the Brownian search and catch mechanism. While rapidly scanning myosin tethered to bead along an actin filament, the binding of myosin to actin is monitored. **a** Schematic of the laser trap used for the experiment. **b** Weakly bound myosin when scanned is either detached from actin rapidly or takes the strong bound state. **c** Typical time course of movement of an optically trapped bead for forward and backward scans. *Arrowheads* indicate transitions from the weak binding to strong binding state. *Left bottom* is an expansion of the time course record for this transition. [Figure modified from Iwaki et al. (2009)]

Laser trap experiments can apply controlled and directional strains at the moment a myosin head binds weakly (Iwaki et al. 2009). In general, a single-headed myosin VI molecule is tethered to an optically trapped polystyrene bead and moved along an actin filament by scanning the bead with the laser trap (Fig. 2a). The scanning speed can be changed to apply a strain at various loading rates up to 3.3 nm/ms. During a scan, a myosin head in the weak binding state attaches and detaches quickly (Fig. 2b). However, should it transition to the strong binding state, the myosin remains bound until after ADP and Pi are released and a subsequent ATP binds (Fig. 2c). The strong binding state was experimentally confirmed by observing binding duration times that were consistent with ADP release and ATP waiting times under loaded conditions. At the maximum loading rate, the weak binding state was observed having short-lived attachments, approximately 1.9 ms, or <2 % the duration of the strong binding state (116 ms).

Strong bindings were more frequently observed at maximum loading rates when a backward strain was applied than when forward strain was (the forward direction of an actin filament is defined as the direction to which most myosin move). However, the frequency of strong binding during the backward scans decreased with the loading rate. Additionally, some fraction of heads had already formed a strong bond before scanning, resulting in strong binding events even during forward scans at low loading rates. Such strong bindings were independent of the speed and direction of the scan and therefore excluded from the analysis. At a rate of 0.2/scan, a weakly bound myosin head proceeded to one of two possible states: detachment or strong binding. We found that 86 % conformed to the strong binding state at the maximum backward loading rate and that the remaining 14 % detached from actin. Consequently, the transition of weakly bound heads to the strong binding state is greatly accelerated by a rapid backward strain. Because the weak- to strong-binding transition is thought to couple with inorganic phosphate (Pi) release from the myosin head, our results strongly suggest that Pi release is accelerated by backward strain.

### Molecular model for accelerated transition

We have proposed a model to explain how strong binding is accelerated in a strain-dependent manner. It has been suggested that Pi is released through an exit route termed the “back door”, and that the closing/opening of the back door is mechanically linked to the opening/closing of the nucleotide-binding pocket, or “front door” (Lawson et al. 2004). The strain dependencies of the ADP release and ATP binding rates have revealed that an external force applied to the head strains the front door. When the neck is pulled backward, the head is bent forward, which closes the front door and opens the back door. Thus, backward strain accelerates Pi release and hence achieves strong binding. However, at the same time the front door is closed, ADP release and ATP binding are suppressed to slow the overall ATP turnover rate. When strained forward, the backdoor is closed or unaffected, resulting in a relatively rare transition to strong binding.

## Molecular model for muscle contraction

Evidence for the Brownian motion of muscle myosin and search-and-catch mechanism from single molecule measurements are the molecular basis for the original Huxley model (Huxley 1957). In this model, thermal motion and preferential binding by myosin connected to a thick filament through springs that are thought responsible for the sliding motion and directional movement of muscle. Based on single molecule measurements, we have constructed a molecular model for muscle contraction (Marcucci and Yanagida 2012). The key of the model is biased Brownian ratchet which can explain how myosin moves along actin monomers when myosin is strongly attached (Rousselet et al. 1994; Esaki et al. 2003; Kitamura et al. 2005). The primitive simulation have demonstrated that Brownian particles move in stepwise manner preferentially in one direction following a periodic and asymmetric potential. In the detached state, myosin moves freely undisturbed by the energy potential that arises with its interaction to actin.

### Attachment and detachment cycles of myosin and actin

**Fig. 3:**
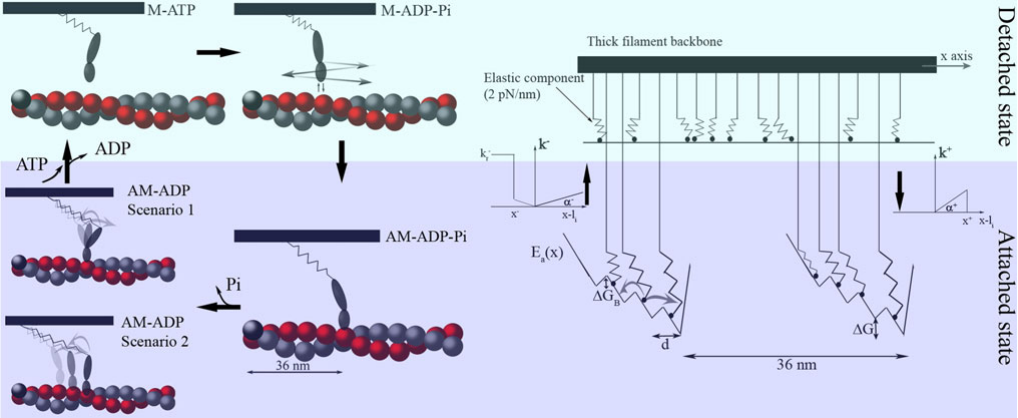
Detachment (*upper panel*) and attachment (*lower panel*) of myosin and actin and the flashing ratchet model (*right*). While myosin detaches and attaches to actin in the ATPase cycle (*left*), myosin heads fluctuate under thermal diffusion while constrained by the elastic element in the detached state and the myosin head during the actomyosin interaction, which results in a flashing Brownian ratchet (*right*). The rates of detachment and attachment are taken from the Huxley model (Huxley 1957) with slight modifications [Figure taken from the paper by Marcucci and Yanagida (2012)]

The detachment and attachment of myosin correlates with the ATPase cycle (Lymn and Taylor 1971) (Fig. 3). In its most minimal form, the cross-bridge cycle can be described by four states: two detached states (M–ATP, M–ADP–Pi) and two attached states (AM–ADP–Pi, AM–ADP). From the M–ATP state, ATP hydrolysis leads to the M–ADP–Pi state, in which myosin takes advantage of Brownian fluctuations to search for the preferred position and create the actomyosin complex (AM–ADP–Pi). The attachment process is driven by the Brownian search-and-catch mechanism. Force generation is thought to correspond to the release of phosphate from the actomyosin complex, leading to the AM–ADP state. Detachment takes place when a new ATP molecule substitutes the exhausted ADP molecule, returning us to the beginning of the cycle (M–ATP).

### Mechanical properties in detached and attached states

The force generating process in the attached state can be described by myosin movement in a local free energy landscape that is based on the biased Brownian movement described above. Local free energy minimums can be interpreted as energy minimums for a myosin conformation such that two scenarios for the force generating process can be considered: (1) the actin and myosin filaments slide past each other due to rotation of the lever arm while the myosin head is firmly attached to the same actin monomer or (2) the myosin head slides along the actin filament. The two competing scenarios share common features that can be used to define the actomyosin complex energy potential. In the Huxley model, they explain how the existence of multiple crossbridge states can generate dynamic muscle behavior. The potential energy is a piecewise linear, multistable potential with four minima equally spaced by distances that correspond to the actin monomer diameter, and is biased in one direction by ΔG. The potential is locally biased but flat on average, repeating itself every 36 nm. Three fundamental parameters are needed to define the potential: the energetic barrier, ΔH, between two minima; the asymmetry of the potential, λ, which represents the ratio of the distances between a minimum and the next maximum and the distances between two minima, d; and the bias or energy barrier, ΔG, between two minima. In the detached state the myosin head is subjected only to thermal fluctuations and to the force generated by the elastic element through which the head is connected to the thick filament.

### Detachment and attachment of myosin and actin

Our model describes the detachment and attachment process using rates assumed as in the original Huxley model rather than using biochemical data. We cannot apply the diffusional approach due to a lack of experimental data for skeletal myosin. The transition between detached and attached states is seen to follow the rate functions depending on the direction of the movement as described in Fig. 3. The attachment rate function comes from experimental results of myosin VI under stress. The detachment rate function is a slightly modified version of our more geometrically detailed potential.

The model was quantitatively tested using two classical experiments for skeletal muscle: fast tension recovery after a small and fast increase of the isometric length and the velocity of contraction against a constant load. When simulating a fast and small change in length (few nanometers per half sarcomere) of a muscle during isometric contraction, typical tension transients can be observed. Initially, an almost instantaneous change from the isometric tension T_0_ to a new value, T_1_, occurs. This is followed by a slower recovery (ms time range) in tension toward T_0_ until a plateau is reached (T_2_). Over a longer time scale, the cross-bridge cycle establishes a fresh population of attached myosins and T_0_ is recovered. The model can simulate the tension versus time traces obtained for different values of length change and gives a good fit of T_1_ and T_2_ values from experimental data. Regarding the force–velocity curve, a muscle fiber bearing a constant load and generating a tension T < T_0_ can contract at a constant velocity in a manner that depends on the load hyperbolically. The simulated V/V_max_ versus T/T_0_ relationship shows very good fitting with the experimental data. Thus, our model based on single molecule measurements can predict standard macroscopic properties of muscle. Thus, whereas previous thermal fluctuation models have used transition rate functions that are based on muscle fiber behavior in a phenomenological fashion to study the above properties, our model directly relates the myosin thermal fluctuation to muscle behavior.

## Conclusion

This article describes how biased Brownian movement and the Brownian search-and-catch mechanism are the basis for muscle contraction. Simulations show that these two mechanisms, as evidenced from single molecule measurements, play a critical role in determining the characteristic properties of muscle. Single molecule measurements can monitor movement at the level of thermal fluctuations and have been used to reveal the mechanism for myosin motor function. The Brownian search ensures that the myosin head samples all possible actin binding sites, while the catch mechanism ensures that myosin only binds strongly at sites that will result in productive powerstrokes. Biased Brownian movement then is the mechanism to actuate random thermal motion into directional movement. These mechanisms are especially efficient for force generation when the motors are assembled to form muscle. Further, it has been demonstrated that myosin motors are typical biological molecular machines that attain their function while under the influence of thermal fluctuations.
